# Association of myocardial hemorrhage and persistent microvascular obstruction with circulating inflammatory biomarkers in STEMI patients

**DOI:** 10.1371/journal.pone.0245684

**Published:** 2021-01-28

**Authors:** Thomas Bochaton, Jules Lassus, Alexandre Paccalet, François Derimay, Gilles Rioufol, Cyril Prieur, Eric Bonnefoy-Cudraz, Claire Crola Da Silva, Hugo Bernelin, Camille Amaz, Sylvie Espanet, Charles de Bourguignon, Nathalie Dufay, Régine Cartier, Pierre Croisille, Michel Ovize, Nathan Mewton

**Affiliations:** 1 INSERM U1060, CarMeN Laboratory, Université de Lyon, Groupement Hospitalier Est, Bron, France; 2 Unité de Soins Intensifs Cardiologiques, Hôpital Louis Pradel et Université Claude Bernard, Hospices Civils de Lyon, Bron, France; 3 Centre Hospitalier Universitaire de Martinique, Université des Antilles, Fort de France, France; 4 Department of Interventional Cardiology, Cardiovascular Hospital and Claude-Bernard University, Bron, France; 5 Centre d’investigation Clinique de Lyon, Hôpital Louis Pradel, Hospices Civils de Lyon, Bron, France; 6 NeuroBioTec, Groupement Hospitalier Est, Hôpital Neurologique Pierre Wertheimer, Bron, France; 7 Centre de biologie Est, Groupement Hospitalier Est, Hospices Civils de Lyon, Bron, France; 8 Université de Lyon, Université Jean-Monnet Saint-Etienne, INSA, Centre National de la Recherche Scientifique, Unité Mixte de Recherche, Creatis, Saint-Etienne, France; 9 Service d’explorations Fonctionnelles Cardiovasculaires, Hôpital Louis Pradel, Hospices Civils de Lyon, Bron, France; Centre National de la Recherche Scientifique, FRANCE

## Abstract

**Introduction:**

Myocardial hemorrhage (IMH) and persistent microvascular obstruction (MVO) are associated with impaired myocardial recovery and adverse clinical outcomes in STEMI patients. However, their relationship with circulating inflammatory biomarkers is unclear in human patients.

**Methods and results:**

Twenty consecutive patients referred for primary percutaneous coronary intervention of first STEMI were included in a prospective study. Blood sampling was performed at admission, 4, 12, 24, 48 hours, 7 and 30 days after reperfusion for inflammatory biomarker (C reactive protein, fibrinogen, interleukin-6 (IL-6) and neutrophils count) assessment. At seven days, cardiovascular magnetic resonance (CMR) was performed for infarct size, MVO and IMH assessment. Median infarct size was 24.6% Interquartile range (IQR) [12.0–43.5] of LV mass and edema was 13.2% IQR [7.7–36.1] of LV mass. IL-6 reached a peak at H24 (5.6 pg/mL interquartile range (IQR) [2.5–17.5]), CRP at H48 (11.7 mg/L IQR [7.1–69.2]), fibrinogen one week after admission (4.4 g/L IQR [3.8–6.7]) and neutrophils at H12 (9.0 G/L IQR [6.5–12.7]). MVO was present in 11 patients (55% of the study population) and hemorrhage in 7 patients (35%). Patients with IMH had significantly higher IL-6, CRP, fibrinogen, and neutrophils levels compared to patients without IMH. Patients with persistent MVO had significantly higher CRP, fibrinogen and neutrophils level compared to patients without MVO, but identical IL-6 kinetics.

**Conclusion:**

In human patients with acute myocardial infarction, intramyocardial hemorrhage appears to have a stronger relationship with inflammatory biomarker release compared to persistent MVO. Attenuating myocardial hemorrhage may be a novel target in future adjunctive STEMI treatments.

## Introduction

Cardiovascular disease, including acute myocardial infarction (MI), is the leading cause of death in Western countries [1,2]. Early reperfusion is currently the most effective treatment to reduce infarct size (IS) resulting from MI [3]. Although reperfusion reduces IS, it causes additional myocardial damage by itself. This process is called ischemia-reperfusion (I/R) injury [4]. Among, the different mechanisms involved in I/R injury, inflammation plays a significant part in the final damage to the ischemic myocardium [5]. The necrosis of ischemic cardiomyocytes triggers an intense inflammatory reaction releasing several mediators, including cytokines [6]. An excessive inflammatory response can cause adverse effects, leading to left ventricular remodeling (LV) and heart failure [5]. Inflammation is assessed through several biomarkers. C-reactive protein (CRP), interleukin-6 (IL-6), fibrinogen and neutrophils count are key inflammatory biomarkers and have been widely studied. They are all related to infarct size and long-term prognosis following MI [7–13]. However, the release kinetics of these biomarkers in reperfused human patients and their relationship with specific components of myocardial injury is poorly known [5,14,15].

Contrast-enhanced cardiovascular magnetic resonance (CMR) is a non-invasive technique that allows the accurate assessment of infarct size, edema, and areas with persistent microvascular obstruction (MVO) or intramyocardial hemorrhage (IMH) [16]. MVO and IMH are independent predictors of adverse LV remodeling and major adverse cardiovascular events (MACE) [16]. In experimental models of ischemia-reperfusion, it was shown that hemoglobin extravasation occurring during MVO and IMH induce a deposit of ferric iron crystals within the infarcted myocardium [17]. These iron crystals induce a sustained pro-inflammatory response [17]. The relationship between post-MI inflammation and the presence of MVO and IMH are not known.

The primary objective of our study was to assess the relationship between markers of CMR severity (IMH and persistent MVO) and the kinetics of the main pro-inflammatory biomarkers (CRP, fibrinogen, IL-6 and neutrophils count) in patients with a first acute ST-elevation myocardial infarction (STEMI) referred for primary percutaneous coronary intervention.

## Methods

### Study population

Consecutive patients admitted with a STEMI and referred for primary percutaneous coronary intervention (PCI) were prospectively enrolled at a single tertiary university hospital. STEMI was defined by the presence of clinical symptoms associated with an ST-elevation of more than 2 mm in two contiguous leads on a standard 12-lead electrocardiogram, or recent Left Bundle Branch Block (LBBB), and presentation within 12-hours of symptom onset according to the European Society of Cardiology [18].

Only patients with a single occluded infarct-related artery (Thrombolysis in Myocardial Infarction grade ≤1) and optimal reperfusion (final TIMI flow ≥2) were included. To obtain a broad sample of infarct size, half of the patient population had a STEMI in the anterior territory and the other half in the inferior territory (LAD and RCA culprit coronary respectively). These selection criteria were set before patient enrollment.

Patients were included if they had: (i) no previous MI, (ii) demonstrated acute single-vessel occlusion; right coronary artery or left coronary artery, (iii) underwent optimal revascularization with TIMI flow ≥ 2 post PCI (iv) had no contraindications to CMR imaging.

Reasons for non-inclusion were as follow: history of prior myocardial infarction, cardiogenic shock, prior cardiac arrest, any contraindication to cardiac CMR (claustrophobia, pacemaker or cardiac defibrillator, known allergy to gadolinium), presence of permanent atrial fibrillation, unconscious patient, severe renal insufficiency (creatinine clearance ≤ 30 ml/min/m2 or renal replacement therapy), long-term immunosuppressive therapy or chronic immunosuppression.

Our institutional review board and Ethics Committee approved this prospective monocentric study. All patients gave written informed consent. The trial design and protocol have been registered ClinicalTrials.gov Identifier: NCT02823886.

### Blood sampling protocol

Seven blood samples were collected for each patient. Venous blood samples were collected at admission to the hospital immediately before PCI and 4 hours, 12 hours, 1 day, 2 days, one week and one month following successful revascularization (H0 H4, H12, H24, H48, 1 week, and 1 month). Each blood sample was centrifuged and treated carefully and stored at the NeuroBioTec Biological Resource Center at -80° C within 4 hours of blood sampling. All samples from our study population were thawed only once to avoid cytokine alteration.

### Biomarkers measurements

IL-6 concentrations were measured by the Human IL-6 Quantikine ELISA Kit (R&D Systems, Minneapolis MN, USA). The limit of detection was 0.7 pg/mL. C-reactive protein (CRP) was determined using immunoturbidimetric methods. Fibrinogen levels was measured in plasma using the Clauss method. Leucocytes and neutrophils count were assessed using fluorescence-activated cell sorting (XN-9000 SYSMEX) at the Hospices Civils de Lyon laboratory.

### Cardiac magnetic resonance protocol

All patients were scanned on a 1.5T CMR MAGNETOM Avantofit system (Siemens, Erlangen, Germany). An intravenous bolus of gadolinium (0.2 mmol/kg body weight; Dotarem, Guerbet, France) was injected by a power injector (Medrad Spectris, Vol-kach, Germany) flushed by 15 ml of saline serum. Late gadolinium enhancement (LGE) was evaluated 10 minutes after contrast injection using a 3D-gradient spoiled TurboFLASH sequence with a selective 180° inversion recovery pre-pulse, in the short axis covering the whole ventricle. LV function at rest was assessed with retrospective ECG-gated steady-state free precession pulse cine sequences (cine TrueFISP) in long and short axis views in the true heart axis. Left ventricular ejection fraction (LVEF), LV end-diastolic volume (EDV), LV end-systolic volume (ESV) and myocardial mass were calculated for each patient with the post-processing software CMR42 (Circle Cardiovascular Imaging Inc., Calgary Canada).

Infarct size was assessed on the 3D data sets by manual planimetry of the LGE images using the post-processing software Osirix (OsiriX Foundation, Geneva, Switzerland). Thus, for all slices infarct absolute mass in grams was measured according to the following formula:

*Infarct mass (g) = ∑ (hyper enhanced area(cm2)) ×slice thickness (cm)×myocardial specific density (1*,*05 g/cm3)*. Relative infarct size (%) was obtained by the ratio of (absolute infarct mass (g)/ LV myocardial mass (g)) ×100.

Edema was quantified in T2 map images using the full-width at half maximum method. (FWHM). Edema was expressed as a percentage of the LV myocardial mass.

Persistent microvascular obstruction was detected on LGE images as hypointense regions in the core of the infarct.

Intramyocardial hemorrhage was identified using T2*-weighted imaging as a hypointense region of reduced signal intensity within the infarcted area, with a T2* value of <20 ms. Delineation of IMH and MVO was performed by two experienced readers (N.M and C.D.B).

### Statistical analysis

Levels of inflammatory biomarkers were identified as not normally distributed. Therefore, those variables were expressed as medians, and interquartile range (IQR) or 95% confident interval and non-parametric tests were used for comparison between groups. At each time-point, comparisons between groups were performed using a Mann-Whitney test. Categorical variables were analyzed using Fisher's exact test. Correlations were done using Pearson correlation method. We used GraphPad Prism 8.4.2. A p-value <0.05 was considered significant.

## Results

### Baseline characteristics

**Fig 1:**
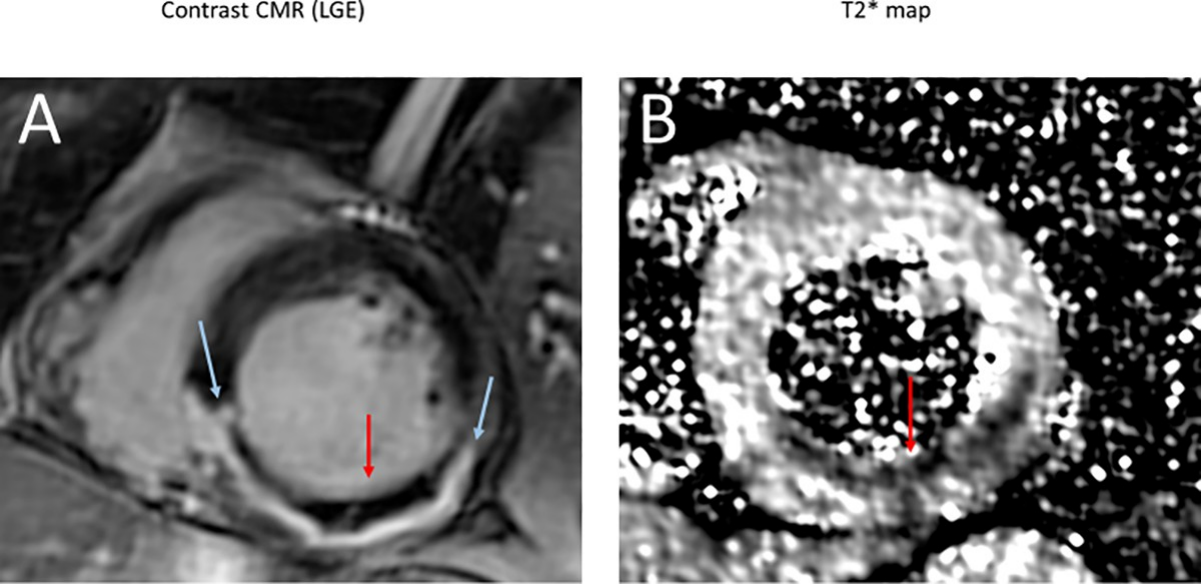
Cardiac magnetic resonance in a patient with an inferior ST-elevation myocardial infarction, treated by primary percutaneous coronary intervention. **A**. Late gadolinium enhancement (LGE) imaging shows the area of microvascular obstruction (red arrow) in the core of inferior transmural infarction (blue arrow). Contrast fails to penetrate the areas of microvascular obstruction and appears as pseudonormal myocardium. **B**. T2* mapping shows an area of intra-myocardial hemorrhage (red arrow).

Twenty consecutive patients referred for primary percutaneous coronary intervention of first anterior or inferior STEMI were included. Patient characteristics at baseline are presented in **Table 1**. Briefly, they were 55±15 years old with 85% males. There were 55% of anterior MI and 45% of inferior MI. CMR imaging was performed for all the patients at a median of 8 days (interquartile range IQR [6–8] days) following PCI. A representative case of a patient with images of myocardial infarction, MVO and IMH is reported in **Fig 1**. Median infarct size was 24.6% of LV mass IQR [12.0–43.5], and edema was 13.2% of LV mass IQR [7.7–36.1]. Intra-myocardial hemorrhage, as defined by T2* imaging was present in 7 patients (35% of the study population). Microvascular obstruction with late gadolinium enhancement CMR was present in 11 patients (55%). CMR infarct parameters are presented in **Table 2**.

**Table 1:** Baseline characteristics of the study population. Values are mentioned as mean ± SD, median with [interquartile range], or absolute number (with percentage).

Age (yr)	55 ± 15
Male Genders no. (%)	17 (85)
Body Mass Index (kg/m^2^)	27 ± 4
Systolic Blood Pressure (mmHg)	122±20
Diastolic Blood Pressure (mmHg)	73±20
Heart Rate (bpm)	74±15
Current Smoker no. (%)	11 (55)
Diabetes mellitus no. (%)	3 (15)
Dyslipidemia no. (%)	4 (20)
Hypertension no. (%)	4 (20)
Ischemia time (min)	163 [129–402]
Killip class at admission no. (%)	
Killip = 1	18 (90)
Killip ≥ 2	2 (10)
Infarct-related artery no. (%)	
Left anterior descending coronary artery no. (%)	11 (55)
Right coronary artery no. (%)	9 (45)

**Table 2:** Cardiac magnetic resonance parameters. Values are mentioned as median with [interquartile range], or absolute number (with percentage).

LV end-diastolic volume (mL)	177.0 [168.0–191.3]
LV end-systolic volume (mL)	90.0 [69.5–105.0]
LV mass (g)	150.0 [123.8–167.3]
LV ejection fraction (%)	53.0 [44.5–57.8]
Infarct Size (% of LV)	24.6 [12.0–43.5]
Edema (% of LV)	13.2 [7.7–36.1]
Presence of MVO no. (%)	11 (55)
Presence of IMH no. (%)	7 (35)

All patients underwent blood sampling at seven-time points (n = 136, n missing = 4): before PCI, 4 hours (H4), 12 hours, 1 day, 2 days, 1 week and 1 month following successful revascularization.

**Fig 2:**
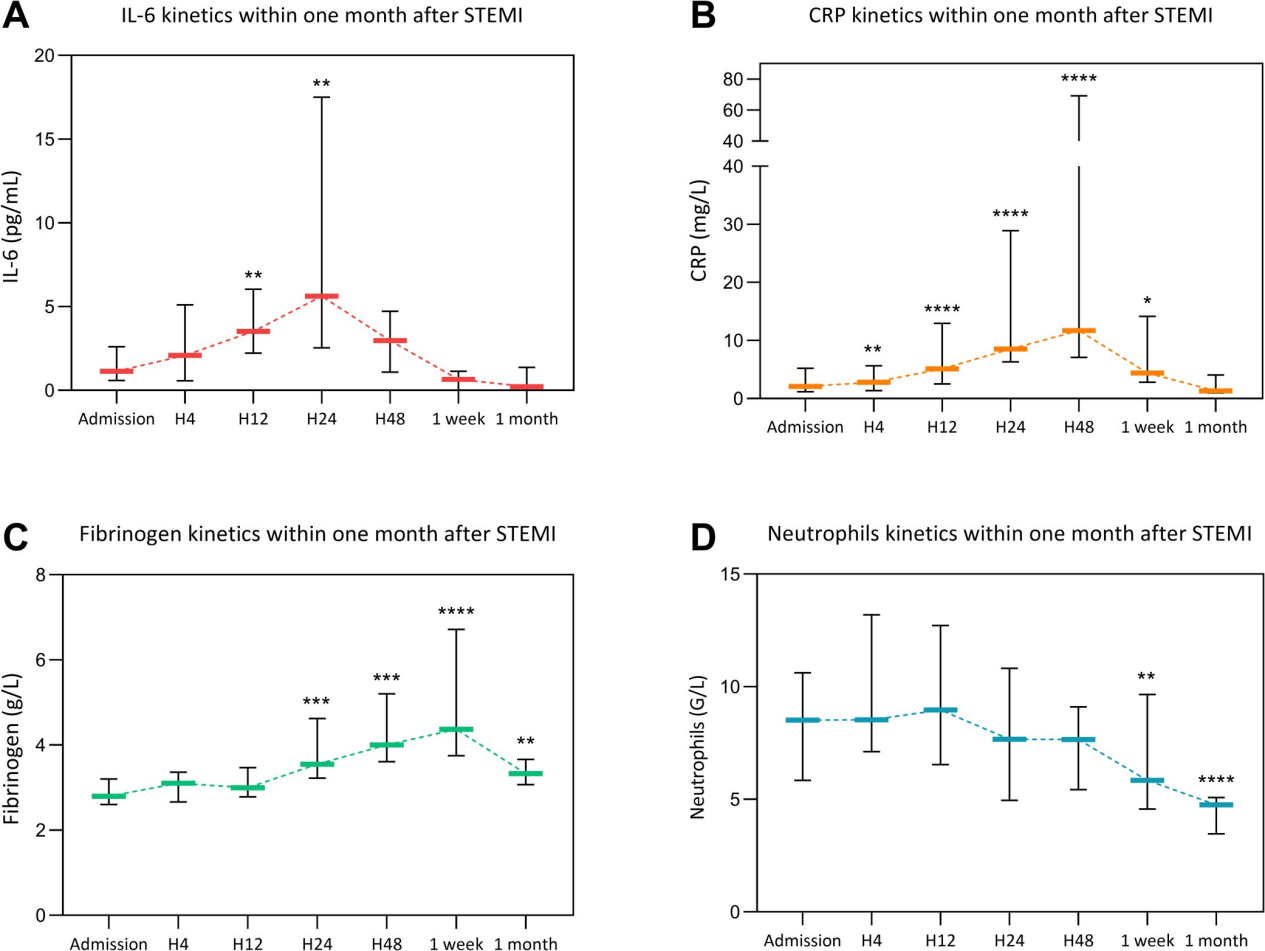
Interleukin-6 (IL-6), C-reactive protein (CRP), fibrinogen and neutrophils count kinetics according within the first month after STEMI. Data are expressed as median with interquartile range (IQR). H4: four hours after admission, H12: twelve hours after admission, H24: twenty-four hours after admission, H48: forty-eight hours after admission. *p<0.05, **p<0.01, ***p<0.001, ****p<0.0001 in comparison with admission level.

Global kinetics of the four biomarkers is presented on **Fig 2**. In our cohort we observed that IL-6 reached a peak at 5.6 pg/mL IQR [2.5–17.5] twenty-four hours after admission (p = 0.002 compared with admission, **Fig 2A**). C-reactive protein reached a peak forty-eight hours after admission at 11.7 mg/L IQR [7.1–69.2] (p<0.0001 compared with admission level, **Fig 2B**). Fibrinogen reached a delayed peak seven days after admission at 4.4 g/L IQR [3.8–6.7] (p<0.0001 compared with admission, **Fig 2C**). Neutrophils reached an early peak twelve hours after admission at 9.0 G/L IQR [6.5–12.7] (p<0.0001 compared with baseline level at one month, **Fig 2D**). The peak of each biomarker was significantly correlated with IS assessed by CMR (r = 0.55, p = 0.01 for CRP, r = 0.64, p = 0.003 for IL-6, r = 0.78, p<0.0001 for fibrinogen and r = 0.67, p = 0.001 for neutrophils). Furthermore, the peak of each biomarker was inversely correlated with LVEF assessed by CMR (r = -0.71, p = 0.0005 for CRP, r = -0.68, p = 0.0009 for IL-6, r = -0.80, p<0.0001 for fibrinogen and r = -0.58, p = 0.007 for neutrophils).

### Association between IMH and inflammatory biomarkers

**Fig 3:**
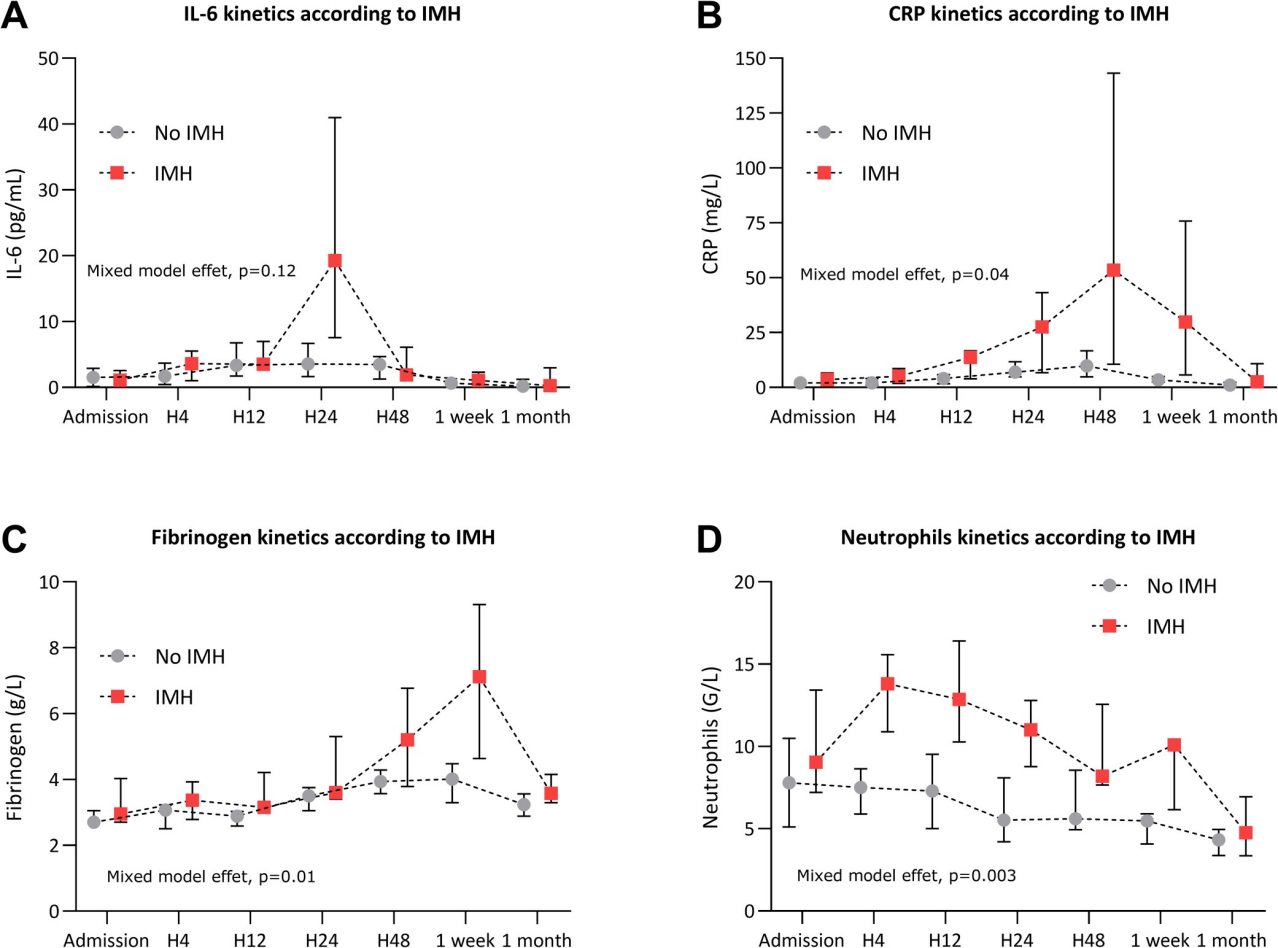
Association between intra-myocardial-hemorrhage (IMH) and inflammatory biomarkers kinetics levels. IL-6 (**A**), CRP (**B**), fibrinogen (**C**) and neutrophils count (**D**) kinetics according to the presence of IMH or no IMH on cardiac magnetic imaging 1 week after STEMI. Data are expressed as median with interquartile range (IQR). IL-6: Interleukin-6, CRP: C-reactive protein. *p<0.05, **p<0.01, ***p<0.001.

The association between inflammatory biomarkers kinetics and IMH are reported in **Fig 3.**

Patients were divided into two groups according to the presence (n = 7/17) or absence (n = 10/17) of IMH. IMH was undetermined for 3 patients. Patients with IMH had significantly higher IL-6 peak levels (at H24) compared to patients without IMH (18.2 pg/mL IQR [5.82–66.5] versus 3.8 pg/mL IQR [1.3–8.0] respectively, p = 0.04) (**Fig 3A**). Patient with IMH had higher CRP and fibrinogen levels one week after the admission for MI compared to patients without IMH (3.4 mg/L IQR [2.5–5.1] versus 29.8 mg/L IQR [5.7–75.8] for CRP, p = 0.01 and 4.0 g/L IQR [3.3–4.5] versus 7.1 g/l [4.6–9.3] for fibrinogen, p = 0.002) (**Fig 3B and 3C**). Patients with IMH had significantly higher neutrophils count levels from H4 to 1 week following MI with a peak at H4 (13.8 G/L IQR [10.9–15.6] versus 7.5 [5.9–8.6], p = 0.0006) (**Fig 3D**).

### Association between persistent MVO and inflammatory biomarkers

**Fig 4:**
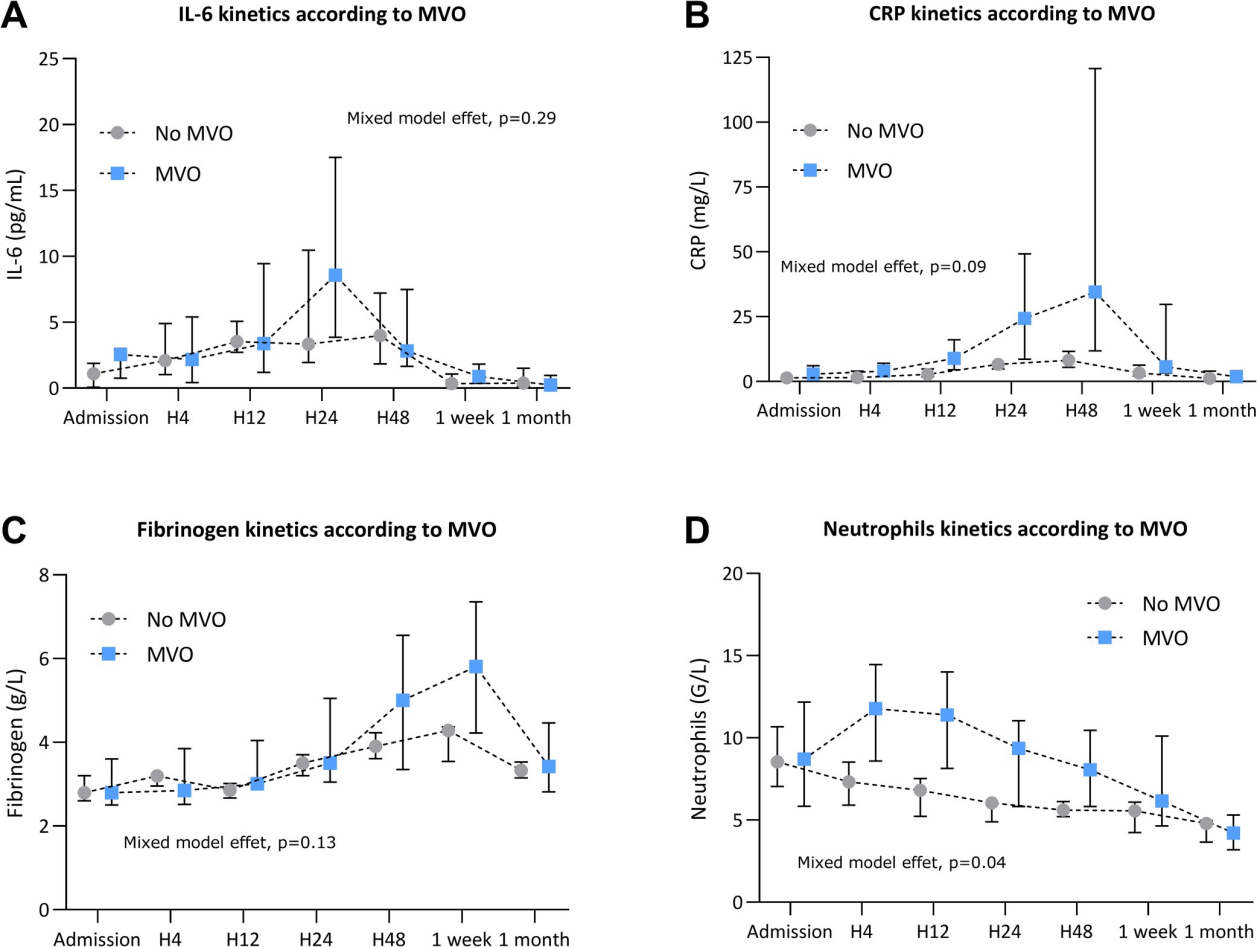
Association between microvascular obstruction (MVO) and inflammatory biomarkers kinetics levels. IL-6 (**A**), CRP (**B**), fibrinogen (**C**) and neutrophils count (**D**) kinetics according to the presence of MVO or no MVO on cardiac magnetic imaging 1 week after STEMI. Data are expressed as median with interquartile range (IQR). IL-6: Interleukin-6, CRP: C-reactive protein. *p<0.05, **p<0.01.

The association between inflammatory biomarkers and MVO is reported in **Fig 4.**

Patients were divided into two groups according to the presence (n = 11/20) or absence (n = 9/20) of MVO. Patients with MVO had similar IL-6 kinetics compared to patients without IMH (**Fig 4A**). However, patients with MVO had higher CRP level at H24 and H48 compared to patients without IMH (respectively 24.3 mg/L IQR [8.6–49.2] versus 6.6 mg/L IQR [4.8–6.9] at H24, p = 0.005 and 34.5 mg/L [11.8–120.7] versus 8.2 mg/L IQR [5.5–11.6] et H48, p = 0.006 (**Fig 4B**). Patients with MVO had higher fibrinogen levels 1 week after MI compared to patients without MVO (5.8 mg/L IQR [4.2–7.4] versus 4.3 mg/L IQR [3.5–4.4], p = 0.045) (**Fig 4C**). They also showed higher neutrophils level count from H4 to H24 following MI with a peak at H4 (11.8 G/L IQR [8.6–14.5] versus 7.3 G/L IQR [5.9–8.5], p = 0.006) (**Fig 4D**).

## Discussion

In a prospective study performed in human patients with reperfused STEMI with inflammation biomarker kinetics over 30 days, our study had three main findings: 1) intramyocardial hemorrhage is significantly related to systemic inflammation with a strong association with pro-inflammatory biomarkers (IL-6, fibrinogen, neutrophils count, CRP); 2) persistent microvascular obstruction was also associated with greater levels of inflammatory biomarkers but this association seemed to be weaker.

### Inflammatory biomarkers at the acute phase of MI

Our study assessed kinetics levels of inflammatory biomarkers within 1 month and to date, it is the most detailed kinetics after STEMI. Liebetrau *et al*. showed an accurate kinetics of inflammatory biomarkers but this kinetics was limited to the first 24 hours and it was evaluated in patient undergoing transcoronary ablation of septal hypertrophy whose pathophysiology differs from STEMI [19]. We observed that all four studied biomarkers had a different peak time point: H4 for neutrophils, H24 for IL-6, H48 for CRP and 1 week for fibrinogen. These results highlight the fact that the knowledge of the precise peak time point is important when studying biomarkers in order not to lose information.

### Association between MVO/IMH and systemic inflammation

This is the first study to systematically study the relationship between different CMR markers (hemorrhage, persistent microvascular obstruction, and edema) with inflammation biomarkers at the acute phase of reperfused ST-elevation myocardial infarction (STEMI). The results of our study show that IMH is significantly associated with a pro-inflammatory pattern.

In patients with STEMI, MVO represents a failure to restore optimal myocardial reperfusion despite re-permeabilization of the epicardial artery by PCI [20]. It is secondary to severe impairment of myocardial microcirculation involving several mechanisms; distal embolization of thrombotic debris, leukocyte infiltration, vasoconstriction, activation of inflammatory pathways and cellular edema [20]. IMH is secondary to the destruction of micro vascularization secondary to hypoxia, inducing extravasation and aggregation of erythrocytes into the tissue extra-vascular space [16,21]. Also, reperfusion is thought to increase leakage from the endothelial junction and damage, thus causing extravasation of red blood cells in the tissue extra-vascular space. MVO is an independent predictive factor of LV adverse remodeling and the occurrence of major cardiovascular adverse events in several clinical and preclinical studies [16,22]. Recent studies have shown that MVO could occur alone but was also frequently associated with IMH [16,21]. The association of these two phenomena in patients appears to carry the worst clinical prognosis with an increased risk of adverse ventricular remodeling, and major cardiac events such as re-hospitalization, heart failure, and death [16,23]. The pathological relationship between these two phenomena is poorly understood. A hypothesis recently suggested by Kali *et al*. [17] in a pre-clinical study is that the degradation products of hemoglobin are transformed into iron ferric crystals in the infarcted myocardium. These iron crystals cause a deleterious prolonged pro-inflammatory burden. Indeed, persistent and excessive inflammation, independent of the size of the infarction, has been suggested to contribute to adverse LV remodeling and an increased risk of future cardiovascular events after MI [5].

Following myocardial infarction, the increased IL-6 synthesis and signaling by myocytes lead to the preservation of heart tissue, in which damage is limited by reducing cell contractility and inducing an anti-apoptotic program [24–26]. However, pre-clinical and clinical studies have shown that excessive IL-6 production is deleterious. Indeed, by inducing an anti-apoptotic program and reducing long-term contractility, the excessive IL-6 secretion may finally lead to adverse LV remodeling and heart failure [27,28]. Also, IL-6 is a major mediator of CRP liver synthesis [29]. Post-STEMI, CRP elevation has been associated with the acute and chronic phase with an increased risk of mortality and cardiovascular events [29,30]. In our study, we found higher CRP levels in patients with IMH. Furthermore, we found a significant association between the presence of IMH and the neutrophil peak level. Neutrophils are involved early in the healing process following reperfusion [31]. However, their excessive recruitment and increased release of ROS (Reactive Oxygen Species) and proteases can be deleterious [31]. The neutrophil rate in post-STEMI is known to be associated with infarct size, adverse LV remodeling, and mortality [32,33]. Finally, we found a significant association between the fibrinogen peak levels, a pro-inflammatory marker associated with infarct size [34,35] and the presence of IMH.

Concerning the presence of MVO, we found a significant association with the CRP peak level. This result is in agreement with the results of Ørn *et al*. [36] showing a significant association between the CRP level at 48 hours and the presence of MVO. Carrick *et a*l. [16] recently showed that MVO is a necessary condition for the occurrence of IMH but that MVO can occur alone. Our results suggest that the presence of MVO alone (without IMH) is associated with a less pro-inflammatory response than MVO associated with IMH. However, this hypothesis must be confirmed by larger studies comparing systemic inflammation between MVO without IMH and MVO with IMH infarctions.

Taken together, our results show that IMH detected by CMR is associated with a systemic pro-inflammatory reaction potentially involved in the poor prognosis associated with this marker. The link between MVO and systemic inflammatory response appears to be weaker.

### Clinical perspective

The clinical perspectives of our study are important. Indeed, the detection of myocardial hemorrhage by CMR may allow targeting patients with the highest inflammatory burden. These patients would be the most likely to benefit from the anti-inflammatory therapies under investigation.

### Limitation

A significant limitation of our study is related to the small sample size of our population. However, our population was homogeneous with infarct sizes, and a frequency of occurrence of MVO and IMH comparable with recent studies [16]. This limited sample size affects the statistical power to demonstrate significant associations between MVO or IMH and biomarkers.

Despite the high number of blood samples taken, considering the short half-life of different markers measured, it is possible we missed the peak values of its marker. Another important limitation is that the biomarkers used to evaluate inflammation are not specific (e.g., CRP, fibrinogen). They reflect the subsequent consequences of the inflammatory activity but do not provide information on local tissue inflammation. Furthermore, if we have identified a relationship between IMH and systemic inflammation, the absence of anatomopathological comparison makes it difficult to describe a causal relationship.

Finally, although the detection of myocardial hemorrhage by T2* sequences is currently the gold standard [16], T2* acquisition was associated with imaging artifacts that limited the quantification of hemorrhage in some patients, and only 85% of the cohort had analyzable T2* data. However, these results are consistent with recent studies evaluating IMH by T2* sequences [16].

## Conclusion

In human patients with acute myocardial infarction, myocardial hemorrhage appears to have the strongest relationship with inflammatory biomarker release compared to persistent MVO or myocardial edema. Attenuating myocardial hemorrhage-induced inflammation may be a novel target in future adjunctive STEMI treatments.

## Supporting information

S1 Fig Association between intra-myocardial-hemorrhage (IMH) and other inflammatory biomarkers kinetics levels. ST2 (A), IL-18 (B), IL-10 (C), TGF-β (D), IL-8 (E), MCP1 (F) kinetics according to the presence of IMH or no IMH on cardiac magnetic imaging 1 week after STEMI. Data are expressed as median with interquartile range (IQR). ST2: Interleukin 1 receptor-like 1, IL-18: Interleukin-18, IL-10: Interleukin-10, TGF-β: Transforming Growth Factor-β, IL-8: Interleukin-8, MCP1: Monocyte Chemoattractant Protein 1. Differences between curves were assessed using a mixed-effect model.
(TIF)

S2 Fig Association between microvascular obstruction (MVO) and other inflammatory biomarkers kinetics levels. ST2 (A), IL-18 (B), IL-10 (C), TGF-β (D), IL-8 (E), MCP1 (F) kinetics according to the presence of MVO or no MVO on cardiac magnetic imaging 1 week after STEMI. Data are expressed as median with interquartile range (IQR). ST2: Interleukin 1 receptor-like 1, IL-18: Interleukin-18, IL-10: Interleukin-10, TGF-β: Transforming Growth Factor-β, IL-8: Interleukin-8, MCP1: Monocyte Chemoattractant Protein 1. Differences between curves were assessed using a mixed-effect model.
(TIF)

S3 Fig ST2 (A), IL-18 (B), IL-10 (C), TGF-β (D), IL-8 (E), MCP1 (F) kinetics within the first month after STEMI. Data are expressed as median with interquartile range (IQR). H4: four hours after admission, H12: twelve hours after admission, H24: twenty-four hours after admission, H48: forty-eight hours after admission.
(TIF)
