# Supplementary figures and images for: Association of myocardial hemorrhage and persistent microvascular obstruction with circulating inflammatory biomarkers in STEMI patients
Source: PLoS One. 2021 Jan 28;16(1):e0245684. doi: 10.1371/journal.pone.0245684 (PMC7842962; doi:10.1371/journal.pone.0245684)

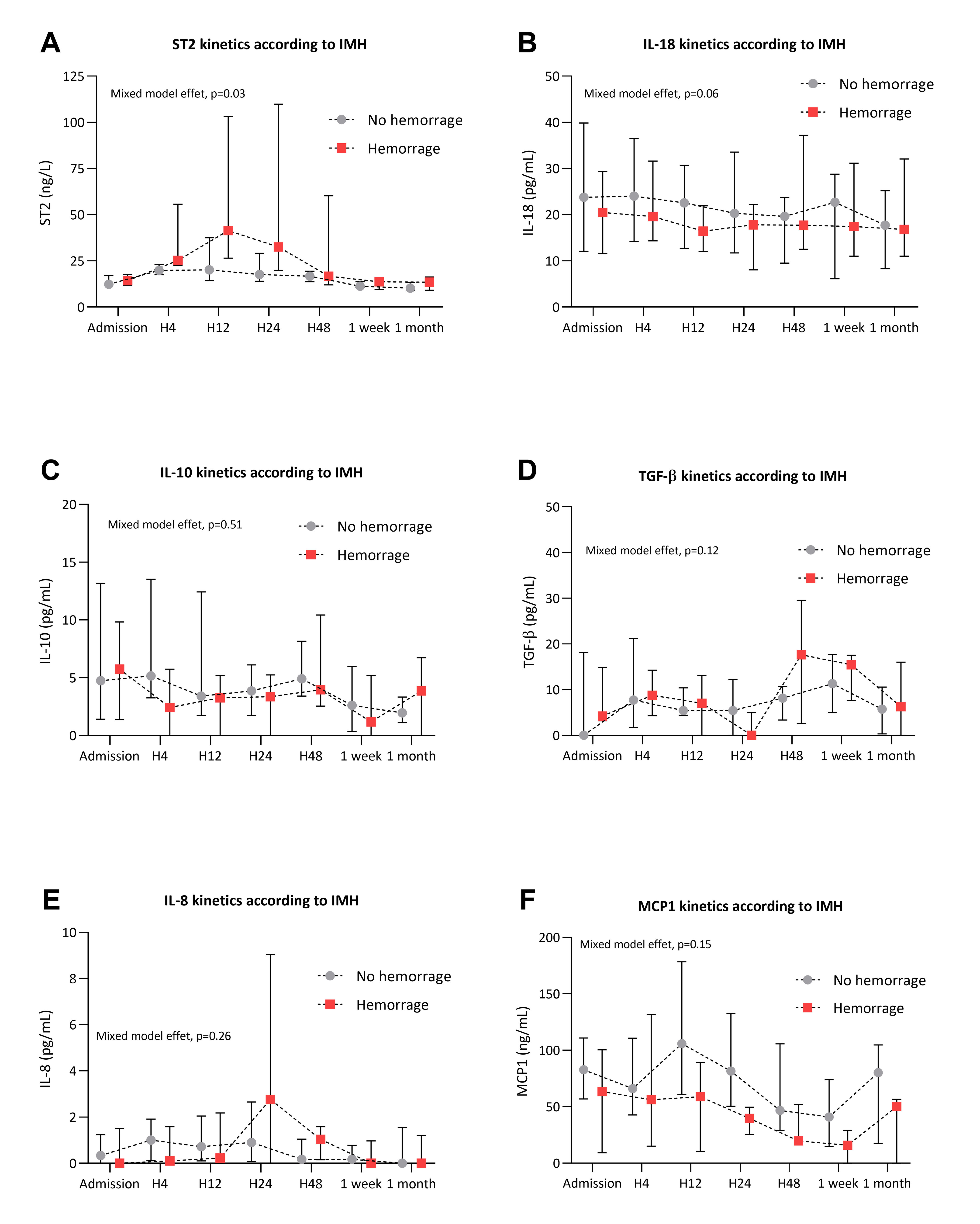

Supplement: S1 Fig — ST2 (A), IL-18 (B), IL-10 (C), TGF-β (D), IL-8 (E), MCP1 (F) kinetics according to the presence of IMH or no IMH on cardiac magnetic imaging 1 week after STEMI. Data are expressed as median with interquartile range (IQR). ST2: Interleukin 1 receptor-like 1, IL-18: Interleukin-18, IL-10: Interleukin-10, TGF-β: Transforming Growth Factor-β, IL-8: Interleukin-8, MCP1: Monocyte Chemoattractant Protein 1. Differences between curves were assessed using a mixed-effect model. (TIF) [file pone.0245684.s001.tif]

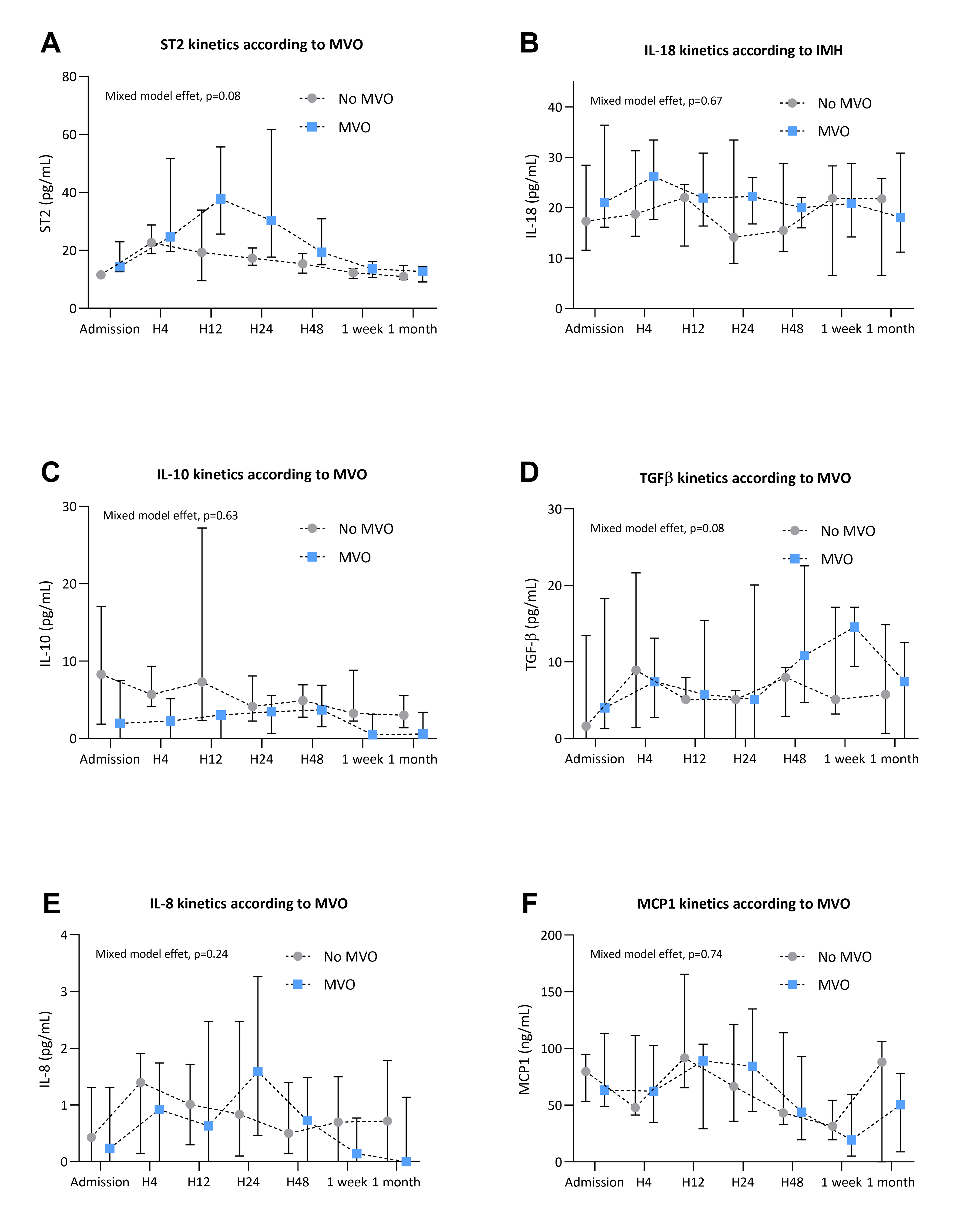

Supplement: S2 Fig — ST2 (A), IL-18 (B), IL-10 (C), TGF-β (D), IL-8 (E), MCP1 (F) kinetics according to the presence of MVO or no MVO on cardiac magnetic imaging 1 week after STEMI. Data are expressed as median with interquartile range (IQR). ST2: Interleukin 1 receptor-like 1, IL-18: Interleukin-18, IL-10: Interleukin-10, TGF-β: Transforming Growth Factor-β, IL-8: Interleukin-8, MCP1: Monocyte Chemoattractant Protein 1. Differences between curves were assessed using a mixed-effect model. (TIF) [file pone.0245684.s002.tif]

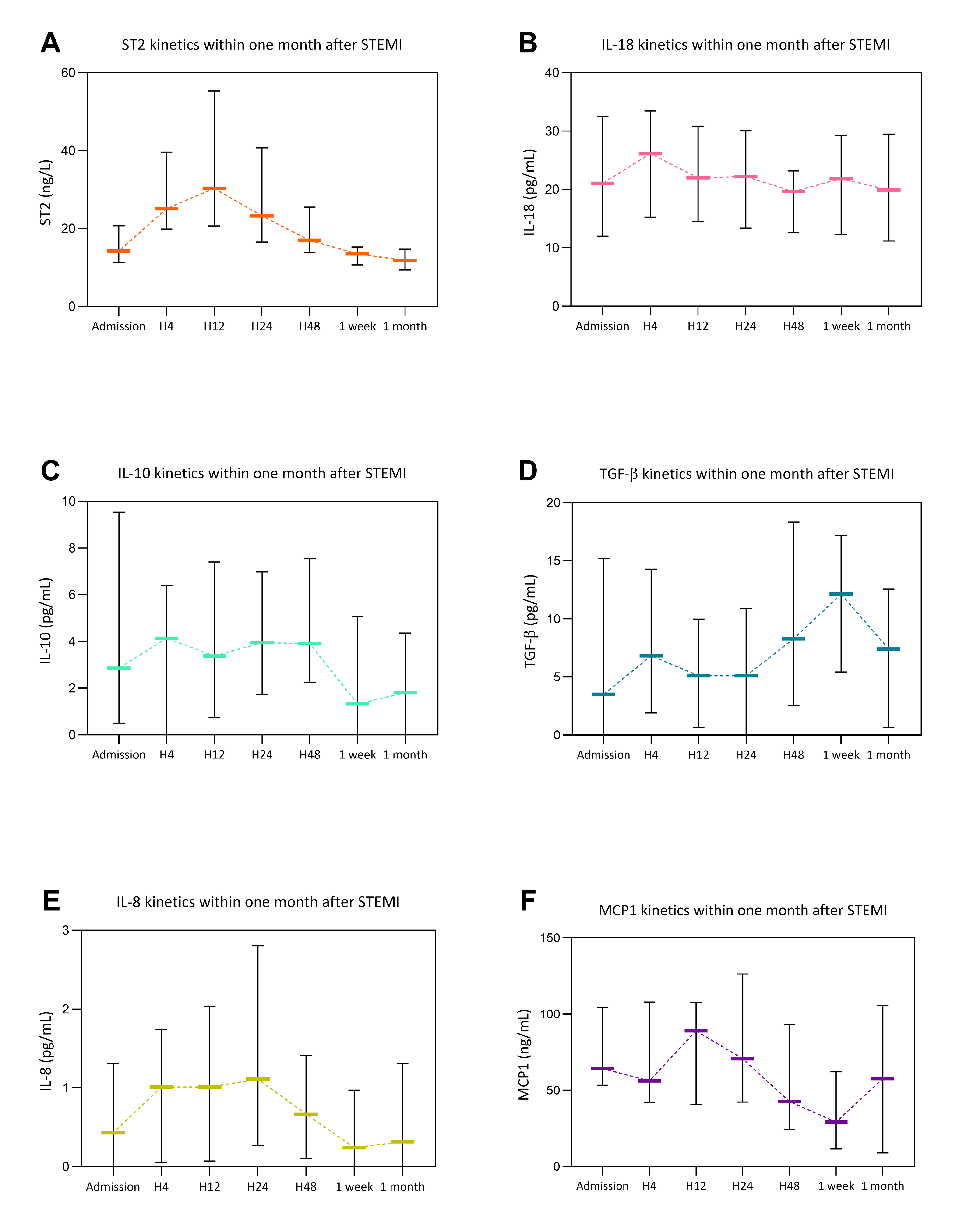

Supplement: S3 Fig — ST2 (A), IL-18 (B), IL-10 (C), TGF-β (D), IL-8 (E), MCP1 (F) kinetics within the first month after STEMI. Data are expressed as median with interquartile range (IQR). H4: four hours after admission, H12: twelve hours after admission, H24: twenty-four hours after admission, H48: forty-eight hours after admission. (TIF) [file pone.0245684.s003.tif]
